# Physical activity and sleep profiles in Finnish men and women

**DOI:** 10.1186/1471-2458-14-82

**Published:** 2014-01-27

**Authors:** Heini Wennman, Erkki Kronholm, Timo Partonen, Asko Tolvanen, Markku Peltonen, Tommi Vasankari, Katja Borodulin

**Affiliations:** 1 National Institute for Health and Welfare, P.O. Box 30, 00271 Helsinki, Finland; 2 University of Jyväskylä, P.O. Box 35, 40014 Jyväskylä, Finland; 3 UKK Institute for Health Promotion and Research, P.O. Box 30, 33501 Tampere, Finland

**Keywords:** Physical activity, Sleep, Chronotype, Latent class analysis, Health behavior

## Abstract

**Background:**

Physical activity (PA) and sleep are related to cardiovascular diseases (CVD) and their risk factors. The interrelationship between these behaviors has been studied, but there remain questions regarding the association of different types of PA, such as occupational, commuting, and leisure time to sleep, including quality, duration and sufficiency. It is also unclear to what extent sleep affects peoples’ PA levels and patterns. Our aim is to investigate the interrelationship between PA and sleep behaviors in the Finnish population, including employment status and gender.

**Methods:**

The study comprised population based data from the FINRISK 2012 Study. A stratified, random sample of 10,000 Finns, 25 to 74 years-old, were sent a questionnaire and an invitation to a health examination. The participation rate was 64% (n = 6,414). Latent class analysis was used to search for different underlying profiles of PA and sleep behavior in men and women, respectively. Models with one through five latent profiles were fitted to the data. Based on fit indicators, a four-class model for men and women, respectively, was decided to be the best fitted model.

**Results:**

Four different profiles of PA and sleep were found in both men and women. The most common profile of men comprised 45% of the total participants, and in women, 47%. These profiles were distinguished by probabilities for high leisure time PA and sleep, subjectively rated as sufficient, as well as sleep duration of 7–7.9 hours. The least common profiles represented 5% (men) and 11% (women) of the population, and were characterized by probabilities for physical inactivity, short sleep, and evening type for women and morning type for men. There was also one profile in both genders characterized by likelihood for both high occupational PA and subjectively experienced insufficient sleep.

**Conclusions:**

The use of latent class analysis in investigating the interrelationship between PA and sleep is a novel perspective. The method provides information on the clustering of behaviors in people and the profiles found suggest an accumulative nature of leisure time PA, and better sleep. Our data also suggest that high levels of occupational PA are associated with shorter and poorer sleep.

## Background

Maintaining or adopting a healthy lifestyle, including high physical activity (PA), enough sleep duration and good sleep quality [1,2] is strongly associated with a lower CVD risk profile [3]. Physical inactivity [4,5] and sedentary behavior [6] are well recognized risk factors for CVD, but too short or long sleep duration and poor sleep quality are suggested to be associated with higher CVD risk as well [7-9].

The interrelationship between PA and sleep behaviors is not fully understood; particularly unclear is to what extent sleep modifies the positive health-effects of PA and vice versa [10]. Epidemiological studies consistently report inverse associations between PA and prevalence of sleep complaints and sleep disturbances [10-12]. Physically active people are reported to have fewer difficulties falling asleep, less nocturnal awakenings, shorter sleep onset latency and less sleep insufficiency, and to feel less tired during the day than inactive persons [13-15].

Intervention studies have shown that sleep quality and total sleep length can be moderately improved by increasing leisure time PA (LTPA) [16,17]. LTPA seems to particularly improve sleep in people who already have sleep complaints or disturbed sleep [18-20]. Short sleep duration and sleep disturbances are observed to be related with less PA [21,22], mobility limitations in the elderly, [23] and to predict future physical inactivity [24].

The role of occupational PA (OPA) on sleep is not that well documented. High work strain and manual work have been linked to poor sleep and higher risk for sleep disturbances [14,25]. The risk for experiencing insufficient sleep has been observed to be higher with increasing occupational physical demands when controlling for LTPA [26].

Circadian rhythm is a factor that may underlie the interrelationship between PA and sleep [17]. Eveningness, or a late chronotype, is directly associated with both sleep complaints [27], shorter sleep during work days [28], and sedentary time [29], whereas morningness, or an early chronotype, has been associated with higher PA [30]. It has also been suggested that regular PA can facilitate a re-entrainment or a change in the phase of the circadian rhythms, a possible advantage, for example, for shift-workers. PA acts as a non-photic zeitgeber for the human biological clock, maybe because of the changes it evokes in body temperature or in the general arousal level [31].

Definitions of short sleep vary, but it has been suggested that different health risks start to increase when habitual sleep duration is equal to or less than 6 hours [32]. The same is true for long sleep at 9 hours or more. However, to what extent self-reported measures of sleep reflect time in bed rather than actual sleep duration is problematic [33]. Long time in bed, which also may be associated with short sleep durations, predicts physical performance decline and incident mobility disability in the elderly [34].

The aim of our study is to search for different sleep and PA profiles, combining information on commuting, occupational and leisure time PA, sleep duration, chronotype, and employment status. Current evidence on associations of PA and sleep is inconclusive on whether and how leisure time PA, occupational PA and commuting PA (CPA) are associated with sleep and chronotype. We assumed that people with higher leisure time PA would also have longer and better sleep than inactive persons but, also, that being physically active in other domains would be related to longer and better sleep.

## Methods

Data for the study were collected in the National FINRISK 2012 Health Study in winter, 2012. The FINRISK Study monitors CVD risk factors in five different regions of Finland. The study protocol follows the recommendations of the WHO MONICA Study and has been repeated in five-year intervals since 1972 [35]. A stratified, random sample of 10,000 Finnish men and women aged 25 to 74 were invited to a health examination and to answer questionnaires assessing health behaviors and health status. The participation rate was 64% (n = 6,424). Participants with any history of CVD (including myocardial infarction, stroke, bypass surgery, angioplasty, angina pectoris, and heart failure) and had missing information on any of the used variables (n = 1,388) were excluded from the analyses. The final sample was 4,470 (1,947 men and 2,523 women). Ethical approval was given by the Coordinating Ethics Committee of the University Hospital District of Helsinki and Uusimaa. Participants gave a written, informed consent.

Employment status was dichotomized into working or not working. Those not working included the retired, unemployed (without work), or homemakers. Subjects reported their sleep duration as average nocturnal sleeping hours. These were grouped into categories: 1.) ≤6 hours, 2.) 6.1-6.9 hours, 3.) 7–7.9 hours, 4.) 8–9 hours, and 5.) ≥9 hours. Time spent napping was calculated as the difference between self-reported nocturnal sleep and 24-hour sleep duration. Napping was categorized into 1.) not napping, 2.) napping for 30 minutes or less, and 3.) napping longer than 30 minutes. Sleep satisfaction was assessed by the question, "Do you think you sleep enough?" and answers were dichotomized into 1.) Yes, always or often, and 2.) No, rarely or I cannot say.

Time in bed was computed for workdays and days off separately, based on self-reported times of going to bed and getting up from bed. The difference between time in bed on workdays and days off was calculated, and this "sleep duration difference" was dichotomized into 1.) 30 minutes or less and 2.) over 30 minutes. Use of sleeping medication was reported as having used: 1.) during the past week, 2.) 1–4 weeks ago, 3.) 1–12 months ago, 4.) Over a year ago, and 5.) Never. Responses were dichotomized into 1.) No, never and 2.) Yes, currently using or has used in past.

LTPA was assessed by the question, "How much do you exercise and stress yourself physically in your leisure time?" Response options were 1.) In my leisure time, I read, watch TV, and work in the household with tasks which do not make me move much and which do not physically tax me, 2.) In my spare time, I walk, cycle or exercise otherwise at least 4 hours per week, excluding travel to work, 3.) In my spare time, I exercise to maintain my physical condition for at least 3 hours per week, 4.) In my spare time, I regularly exercise several times a week in competitive sports or other heavy sports. Answers were categorized into 1.) no LTPA, 2.) light LTPA, and 3.) moderate to high LTPA that includes responses three and four. OPA was assessed with one question: "How demanding is your work physically?" with response options: 1.) I work mainly sitting, 2.) I walk quite much in work but do not have to lift or carry heavy objects, 3.) I have to walk or lift much and 4.) I do heavy manual labor. Answers were categorized as 1.) No OPA, 2.) Light OPA and 3.) Moderate to High OPA that included responses three and four. CPA was assessed by the question: "How many minutes do you walk, ride on a bicycle or otherwise exercise to get to work?" There were six response options: 1.) I do not work or I use only a motorized vehicle, 2.) Less than 15 minutes daily, 3.) 15–29 minutes daily, 4.) 30–44 minutes daily, 5.) 45–59 minutes daily and 6.) over an hour daily. Answers were grouped into three categories: 1.) Not physically active in commuting, 2.) less than 30 minutes CPA, and 3.) 30 minutes or more CPA. A sum of total screen-time hours was computed based on the question, "How many hours on average do you sit on a weekday at home watching television or videos or at a computer?" The sum was categorized into gender-specific thirds.

Chronotype was initially assessed by 6 questions on morningness-eveningness preference, modified from the Horne-Östberg morningness-eveningness questionnaire [36]. The shortened version of this questionnaire does not have validated cutoff points for different chronotype groups, so we decided to use an empirical operationalization using latent class analysis (LCA) on data from the 6 original items.

### Statistical analyses

Statistical analyses for investigating the interrelationship between PA and sleep were performed in SAS version 9.3 (SAS Institute Inc., Cary, NC), and included binomial logistic regression and LCA. LCA is a latent variable model that serves to look for underlying subtypes of individuals with the same kind of individual characteristics in the sample [37,38]. The probability for an individual to belong to each class is estimated based on item-response probabilities, conditional on latent class. The number of latent classes represents the number of different subpopulation clusters in the sample [38-40].

We were interested in identifying latent PA and sleep classes in the population based on class specific item-response probabilities on six different sleep items and four different PA items. First, all sleep items were regressed on dichotomized PA items and those predicting PA were chosen to be included in LCA. All items in LCA were categorical. The response categories as well as the proportions of people selecting each response alternative are presented in Table 1. We used PROC LCA [41] command procedure to estimate model parameters. Data on questionnaire items are assumed to be missing at random and are handled within an expectation-maximization algorithm. The expectation-maximization algorithm produces full information maximum likelihood estimates for parameters [39]. Models with one through five classes were fitted in order to find the optimal number of latent classes. Measurement invariance between genders was tested by fitting a model where all item response probabilities were free to vary, and another where item response probabilities were constrained to be equal across genders. The difference in G^2^ statistics was statistically significant, suggesting that measurement invariance could not be assumed to hold across genders. Thus separate models for genders were created.

**Table 1:** Observed total frequency (N) and relative response proportion (%) of men and women

		**Total N**	**Men (n,%)**	**Women (n,%)**	**p**
**Leisure time PA**		4461			
	Not active		341 (17.5%)	516 (20.5%)	0.0011
	Lightly active		939 (48.2%)	1261 (50%)	
	Moderately to highly active		664 (34.1%)	740 (29.3%)	
**Occupational PA**		4433			
	Not active		1160 (59.6%)	1524 (60.4%)	<0.0001
	Light active		317 (16.3%)	608 (24.1%)	
	Moderately to highly active		454 (23.3%)	370 (14.7%)	
**Commuting PA**		4302			
	Inactive		1275 (65.5%)	1443 (57.2%)	<0.0001
	≤ 29minutes /day		444 (10.1%)	655 (13.9%)	
	≥ 30 minutes /day		149 (7.7%)	336 (13.3%)	
**Screentime**		4345			
	Lowest third		0-2.17 hrs	0-2.0 hrs	0.0002
	Mid third		2.17 ≥ 3.9 hrs	2.0 ≥ 3.0 hrs	
	Highest third		>3.9 hrs	>3.0 hrs	
**Nocturnal sleep**		4372			
	≤ 6 hrs		84 (4.3%)	93 (3.7%)	<0.0001
	6.1-6.9 hrs		298 (15.3%)	304 (12.1%)	
	7-7.9 hrs		756 (38.8%)	884 (35.0%)	
	8-9 hrs		719 (36.9%)	1079 (42.8%)	
	≥ 9 hrs		53 (2.7%)	102 (4.0%)	
**Do you sleep enough?**		4421			
	Yes		1600 (82.2%)	1986 (78.7%)	0.0090
	No		331 (17.0%)	504 (20.0%)	
**Chronotype preference**		4137			
	Morning		598 (30.7%)	665 (26.4%)	<0.0001
	More morning		490 (25.2%)	560 (22.2%)	
	More evening		606 (31.1%)	799 (31.7%)	
	Evening		253 (13%)	498 (19.7%)	
**Do you use sleeping medication?**		4187			
	Yes		370 (19.0%)	710 (28.14%)	<0.0001
	No, never		1446 (74.3%)	1661 (65.8%)	
**Sleep duration difference**		4030			
	≤ 0.5 hrs		886 (45.5%)	1168 (46.3%)	0.8114
	> 0.5 hrs		845 (43.4%)	1131 (44.8%)	
**Napping**		4355			
	No		1177 (60.5%)	1713 (67.9%)	<0.0001
	≤ 0.5 hrs		330 (17.0%)	358 (14.2%)	
	> 0.5 hrs		398 (20.4%)	379 (15.0%)	

PA: Physical activity, p: p-value for chi-square test.

The choice of the best model was based on the Bayesian information criterion (BIC), where the smallest value represents the most optimal model fit, taking into account the sample size and number of freely estimated parameters. We also looked at entropy and identification percentage, of which entropy close to 1 and an identification percentage closer to 100% describes better model homogeneity. Average posterior probabilities that describe class separation were also considered. As a rule of thumb, average posterior probabilities over 0.7 indicate good class separation [37]. Our decision stood between the three and four class models, and, regarding all the criteria mentioned above as well as interpretability and prevalence of the latent classes, we chose the four class model.

## Results

Mean values and distributions of descriptive factors by gender are presented in Table 2. The mean age of the population was 52 years for men and 51 years for women. Employment rate was almost 65% in both genders. Men slept on average 7.4 hours while women slept 7.6 hours per night.

**Table 2:** Descriptive variables of men and women as mean (±SD) values and observed (percentage) frequencies

	**Men**	**Women**	**p**
**Age (years)**	52 (± 14)	51 (± 14)	0.0009
**Height (m)**	1.77 (± 0.1)	1.63 (± 0.1)	<0.0001
**Weight (kg)**	85.4 (± 14.2)	71.1 (± 14.3)	<0.0001
**BMI (kg/m**^**2**^**)**	27.2 (± 4.2)	26.7 (± 5.4)	<0.0001
**Nocturnal sleep (hours)**	7.4 (± 1.0)	7.6 (± 1.0)	<0.0001
**Education (years) All**	13.0 (± 3.9)	13.9 (± 4.1)	<0.0001
Lowest third	9.5 (± 2.0)	10.4 (± 2.5)	
Middle third	12.5 (± 2.2)	13.3 (± 2.5)	
Highest third	16.9 (± 2.7)	17.7 (± 3.1)	
**Smoking**			
Not smoking	944 (48.5%)	1641 (65.0%)	<0.0001
Quitted	613 (31.5%)	495 (19.6%)	
Smoking	374 (19.2%)	371 (14.7%)	
**Employment status**			
Not employed or retired	675 (34.7%)	883 (35.0%)	0.9031
Employed	1258 (64.6%)	1633 (64.7%)	

p: p-value for t-test between genders (age, height, weight, body mass index, nocturnal sleep and educational years) and chi-square (smoking and employment).

### LCA for chronotype

Chronotype was determined by fitting latent class models with one to six classes with the data on the six original chronotype questions. Based on the Bayesian information criteria (BIC) and interpretability of the models, a 4 class latent model was selected. The first chronotype class (17.5%) was characterized by likelihood for morning tiredness and self-reported eveningness, and a preference to work hours between 14 and 16 o´clock. This group was called "evening types". The second chronotype class (26.9%) was characterized by likelihood for strong morning alertness and self-reported morningness, called the "morning types". The third latent chronotype class (25.3%) was characterized by likelihood for self-reported more morningness than eveningness, with fair morning alertness. This group was called "more-morning-than-evening types". The fourth chronotype class (30.2%) was characterized by likelihood for self-reported eveningness more than morningness and poor morning alertness, but still feeling quite rested and able to easily to get up in the morning. These were called the "more-evening-than-morning types". All 4 latent chronotype classes were well identified and the average posterior probabilities were over 0.8 respectively, describing good class separation [37]. Furthermore, the four classes were tested in the data by comparing the distributions in midpoint of sleep, a suggested measure of chronotypes [28]. The four classes differed, as was expected, in their midpoint of sleep as follows: morningtypes, 2:49; more-morning-than-evening types, 3:14; more-evening-than-morning types, 3:45; and evening types, 4:25. This supported the validity of chronotype classification obtained by the LCA, which was used in analyses for this study.

### LCA for PA and sleep profiles

**Figure 1:**
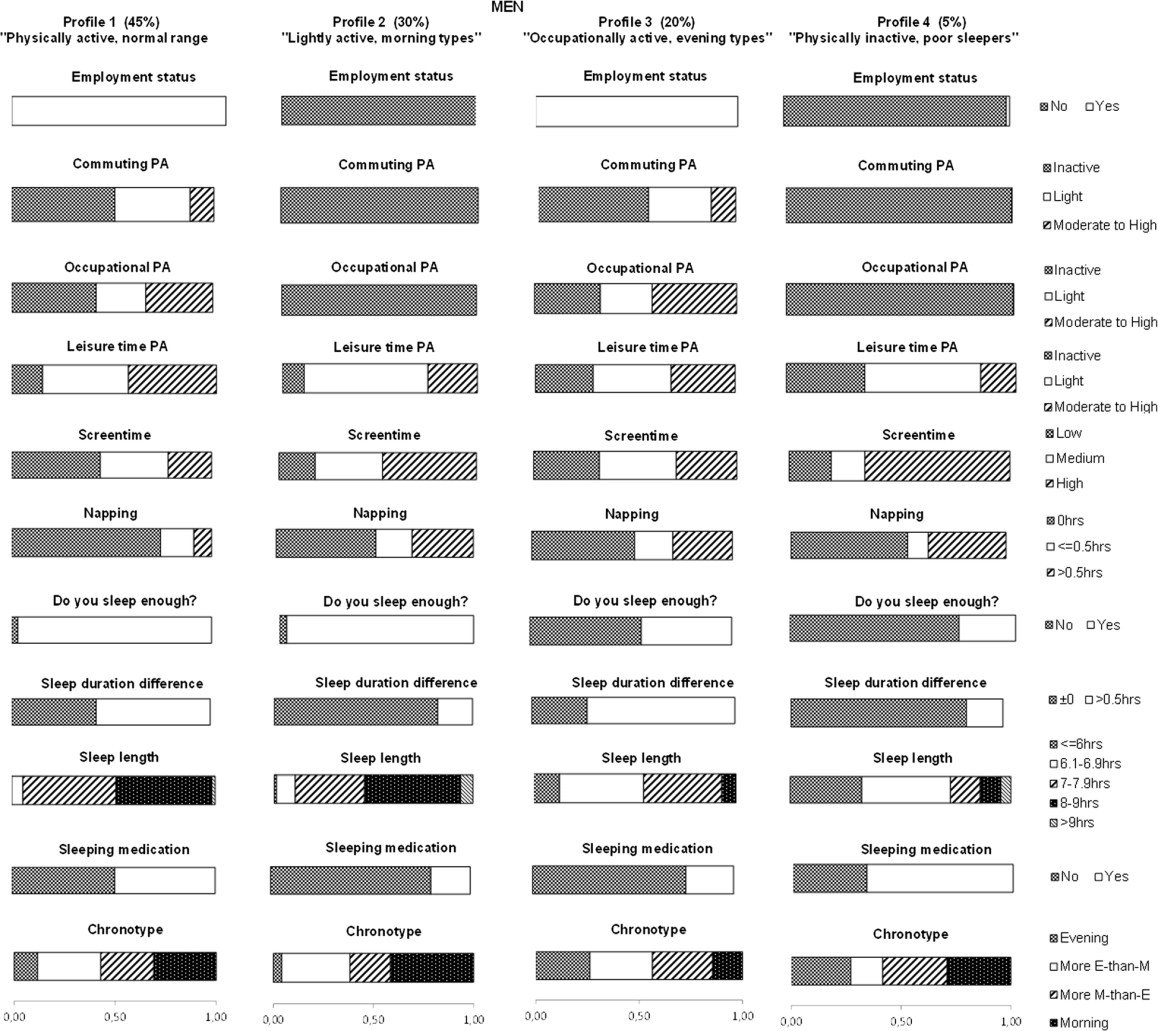
**PA and sleep profiles in men.** The bars represent the response probabilities (0 to 1.0) for each item in respective profile. The prevalence (%) of each profile is given in top. More E-than-M type = More evening-than-morning type. More M-than-E type = More morning-than-evening type.

**Figure 2:**
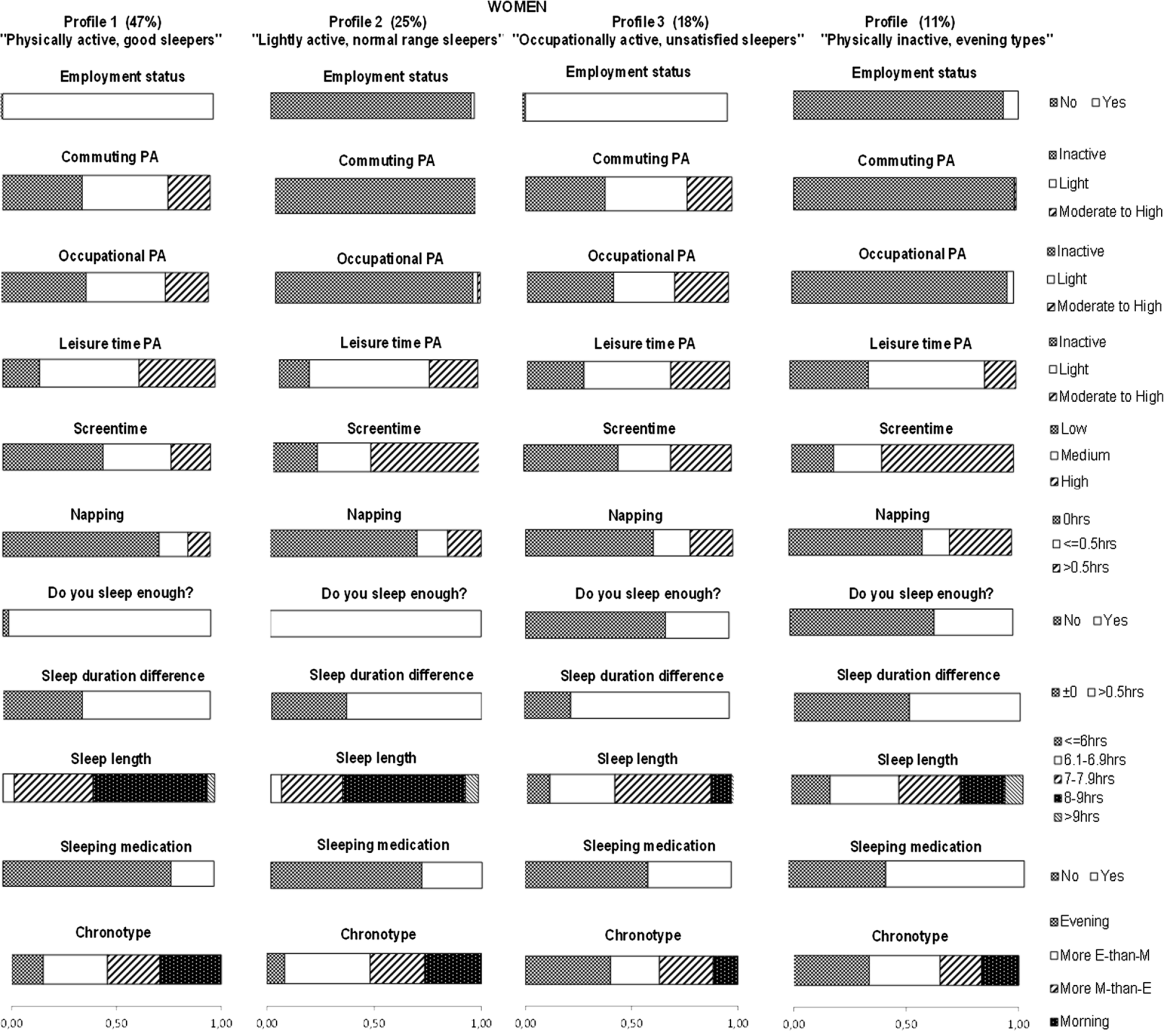
**PA and sleep profiles in women.** The bars represent the response probabilities (0 to 1.0) for each item in respective profile. The prevalence (%) of each profile is given in top. More E-than-M type = More evening-than-morning type. More M-than-E type = More morning-than-evening type.

The LCA with PA and sleep items resulted in four latent classes of different PA and sleep behavior profiles for both men and women. Table 3 shows the essential model identification criteria for each model in women and men. All profiles and item response probabilities are presented in Figure 1 for men and Figure 2 for women.

**Table 3:** Identification criteria for each LCA model with different number of latent classes by gender

**Nr of latent classes**	**Log likelihood**	**Degrees of freedom**	**BIC**	**Entropy**
**MEN**				
**1**	-18418.4	77738.0	9612.7	1.0
**2**	-17288.3	77716.0	7519.1	1.0
**3**	-17125.4	77694.0	7360.1	0.8
**4**	-17019.5	77672.0	7314.7*	0.8
**5**	-16953.7	77650.0	7349.8	0.8
**WOMEN**				
**1**	-24335.1	77738.0	12238.9	1.0
**2**	-22916.2	77716.0	9573.5	0.9
**3**	-22683.7	77694.0	9280.8	0.8
**4**	-22549.2	77672.0	9184.1*	0.8
**5**	-22474.8	77650.0	9207.6	0.8

* = The average posterior probabilities for the different classes in this model varied for men between 0.85 and 0.97 and for women between 0.87 and 0.91.

In men, the first and most prevalent profile (45%) is characterized by working status and highest likelihoods for light CPA, moderate-to-high LTPA, low screen-time, no napping and being satisfied with their sleep. In addition, men in this profile are likely to be inactive in commuting, sleep 7–9 hours, sleep longer during free days, and not to use sleeping medication. This profile is called "physically active, normal range sleepers".

In men, the second profile (30.2%) is characterized by not working and highest probabilities for light LTPA, not sleeping longer during days off, to be satisfied with one’s sleep, and being definitely morning type. In addition, in this profile, it is likely for men to have high screen-time, to not take naps, sleep for 8–9 hours, and to not use sleeping medication. This profile is called "lightly active, morning types with normal range sleep".

The third profile in men (20%) is characterized by working and highest likelihood for moderate-to-high OPA, medium screen-time, sleeping longer during days off, and being evening or more-evening-than-morning type. Also, men in this profile have probably no CPA, are not satisfied with their sleep, sleep more than 6 hours but less than 7 hours, but are more likely to not be using sleeping medication. This profile is called "occupationally active, evening type short sleepers".

The least prevalent, fourth profile in men (4.8%), is characterized by not working and the highest likelihood for inactivity in leisure time, high screen-time, napping over 30 minutes, not being satisfied with their sleep, not sleeping longer during days off, sleeping for 6 hours or less, and using sleeping medication. Men in this profile are also likely to be morning or more-morning-than-evening types. This profile is called "physically inactive, poor sleepers".

The most prevalent, first profile in women (47%) is characterized by working status and highest likelihoods of light OPA, moderate-to-high LTPA, medium screen-time, no napping, being satisfied with their sleep, and not using sleeping medication. In addition, women in this profile are likely to be lightly active in commuting, sleep longer during days off, and sleep for 8–9 hours. This profile is called "physically active, good sleepers".

The second profile (24.8%) in women is characterized by not working and highest probabilities for light LTPA, being satisfied with their sleep, not sleeping longer during days off, and sleeping for 8–9 hours. Women in this profile also are likely to have high screen-time, to not take naps and to not use sleeping medication. This profile is called "lightly active, normal range sleepers".

The third profile in women (17.7%) is characterized by working and the highest likelihood of moderate-to-high OPA, not being satisfied with their sleep, sleeping longer during days off, and eveningness. Also, women in this profile are likely to have light LTPA, low screen-time, to not take naps or not use sleeping medication, and to sleep for 7–7.9 hours. This profile is called "occupationally active, unsatisfied evening type sleepers".

The least prevalent, fourth profile in women (10.7%) is characterized by not working and highest probability for inactivity in leisure time, high screen-time, napping over 30 minutes, sleeping for 6 hours or less, and using sleeping medication. Also, in this profile, women are likely to be evening or more-evening-than-morning types. This profile is called "inactive, evening type short sleepers".

In men, the mean age (with standard deviation, SD), respectively for profiles 1–4 was: 46.6 (11.6), 64.6 (8.4), 44.5 (11.1), and 59.5 (10.9) years. The corresponding mean age in women’s profiles 1 to 4 was: 45.5 (11.2), 61.6 (12.2), 45.2 (10.9), and 58.8 (13.4) years. Mean years of schooling for men in profiles 1 to 4 was: 14.2 (3.6), 11.2 (3.8), 13.7 (3.5), and 11.3 (3.8), and for women in profiles 1 to 4: 14.9 (3.8), 12.1 (4.1), 14.8 (3.4), and 12.3 (4.2) years. The mean age differed significantly between all profiles in men. Profile 2 was the oldest and profile 3 the youngest. In women, profile 2 was significantly older than the other profiles and profile 4 was significantly older than profiles 1 and 3 but there was no significant difference between the two youngest profiles 1 and 3. In both men and women the mean educational years were significantly higher in profiles 1 and 3 compared to profiles 2 and 4. However, there was no statistically significant difference in educational years between profiles 1 and 3 or profiles 2 and 4, neither in men nor in women.

## Discussion

The four PA and sleep profiles found in men and women implicate, as expected, that higher LTPA and better sleep are interrelated. Our profiles suggest an accumulative nature of risk behaviors as the probabilities for physical inactivity in leisure time, subjective feeling of not sleeping enough, short sleep, and evening type are high in the same profiles. In the most common profiles, likelihood for high LTPA co-occurs with subjective sleep satisfaction and normal range sleep. In the least common profiles, inactivity and high screen-time occur together with poor sleep. To our knowledge, this is the first study that combines a variety of PA and sleep behaviors in the same latent class model in order to investigate the interrelationship of these behaviors.

Sleep satisfaction is an item clearly separating profiles in our study. Both the two "working" and "not-working" profiles differ significantly in terms of sleep satisfaction. In women, it is very likely that people in profiles 3 and 4 report dissatisfaction with their sleep. In men, on the other hand, the probability for dissatisfaction with sleep is only slightly higher than the probability for being satisfied with one’s sleep in profile 3, suggesting a higher uncertainty regarding this item within this profile. Whether the subjective experience of sleep is influenced directly by sleep duration or by other factors, it cannot be concluded, but the subjective sleep satisfaction may be an important link between favorable health behaviors. The subjective feeling about insufficient sleep may be as important as true sleep duration or a diagnosed sleep disorder when studying health outcomes of short sleep. It has been observed that the greater the sleep satisfaction, rather than the subjectively or objectively measured sleep duration, the greater the quality of wellbeing is [42], and that self-reported insufficient sleep is related to an unhealthier lifestyle, including physical inactivity [43].

Men and women had somewhat different PA and sleep profiles, and it is not appropriate to compare them directly. Based on the test of measurement invariance as well as fundamental differences in sleep and PA behaviors between men and women, it was decided that genders are analyzed separately, instead of constraining item response probabilities to be equal across genders and thus having the same profiles for both groups. Gender profiles differ in likelihoods for CPA, longer sleep, napping and chronotype. Along with our study, it has been earlier reported that Finnish women report more CPA [44], and longer sleep duration [45], but also more insufficient sleep [46], and more symptoms of insomnia [24] than Finnish men. Prevalence of evening chronotype is higher among Finnish women while morning chronotype is more frequent among men [27].

Except for differences between genders, it is also evident that two profiles are characterized by the employed, and that two comprise the not employed. It is interesting to notice that of the two "not-working" profiles, the likelihoods for LTPA and longer, sufficient sleep occur in the same profile. This implicates a positive association between PA and sleep quality also among the not-working people, which is in agreement with Brassington and Hicks [47], who reported that those older, retired men and women, having regular aerobic exercise, sleep longer, take shorter naps, and have a better overall sleep quality and better daytime functional ability than their counterparts who are not regularly physically active. Studies have reported that those elderly people who are retired or not employed have different sleep and PA behaviors than younger or working people. Both short and long sleep is more likely in older people [48]. Insomnia symptoms are reported to occur more frequently in unemployed or retired [45,49], and morning preference is more common with higher age [27,28].

According to our profiles, people with moderate to high OPA are also more likely to sleep less and not be satisfied with their sleep, often trying to make up for sleep debt from work days during days off. This finding is in agreement with previous findings that physically demanding work can be harmful for sleep [14,50]. It has also been reported that there are big differences in sleep timing between workdays and days off in employed people, resulting in accumulation of sleep-debt over the working week [28,51]. This is evident in all "working" profiles, where the likelihood for sleeping over 30 minutes more during days off is high. Chronotype has been suggested to be linked to these fluctuations in sleep duration between workdays and free-days, arguably because later chronotype have more sleep-debt over the week and more "oversleep" in days off [28]. Indeed, in profiles with likelihood for OPA, especially in women, the likelihood for evening chronotype is higher than the likelihood for morning type. We do not know to what degree, for example, shift-work influences this particular profile, but it is possible that working evenings and nights can explain the higher likelihood for OPA, short sleep, napping, and late chronotype in these profiles. Shift-work is reported to have a positive association with the frequency of sleep complaints in Finnish men working in industry, transport, and traffic. Different shift schedules and combinations have different associations with PA and sleep behavior, but not all are disadvantageous [52]. In Finns, working, in general, is related to shorter and insufficient sleep [46,53] and the prevalence of sleep problems among employed people has increased over the past years [53].

### Strengths and limitations

All data are self-reported, so it is not possible to establish precise amounts of PA or time asleep. In addition, we cannot make any exact prediction of whether people reach the currently recommended PA levels or not. The question about LTPA does not differentiate between separate forms of PA, and the information is only sufficient enough to distinguish inactive from active and highly active. We did not have the information on shift-work in our data and thus could not include it in our analyses. The assessment of chronotype was based on a commonly used instrument, only on self-reports, which is considered a limitation. Because of a cross-sectional design, we cannot conclude on causal relationships between sleep and PA. Still, as a strength of the LCA method, our results give valuable information about the clustering of PA and sleep behaviors that is not possible to attain using more traditional statistical methods such as regression analysis. In a recent review, it was reported that, most often, co-occurrence of multiple risk behaviors is studied using index scores or prevalence rates, and that LCA was used in only two of the 50 reviewed studies [54]. LCA is a person-oriented model that does not allow for conclusions to be drawn about any linear relations between the observed variables, but emphasizes the individual in particular. The use of latent class techniques allows the possibility to organize people into meaningful, homogenous subgroups based on an array of observed data [38,54]. In LCA, subjective interpretation plays a significant role. For example, is the decision of the appropriate number of classes in LCA dependent also upon the investigator’s judgment and substance knowledge, and not solely on fit indices [38]. In this study, it can be argued that genders should be included in the same LCA, as they have very similar profiles. This is also a judgment call that is based on statistical or interpretational or both types of evidence. This time, we decided on keeping genders separate. The strength of our study is the large, population-based health survey data that allows this kind of profile analysis.

## Conclusion

We found four PA and sleep profiles in men and women to represent the Finnish adult population. Our data suggest that sleep and PA are related and that normal, sufficient sleep is associated with higher LTPA, while high inactivity and poor sleep appear hand in hand. Furthermore, high OPA is associated with the likelihood for shorter and insufficient sleep. More studies investigating the clustering of health-behaviors are important to elucidate gender differences and to understand how health-behaviors are linked to each other. Health and lifestyle counseling programs, as well as political decisions, can adapt information from these profiles to better focus their actions.

## Competing interests

The authors declare that they have no competing interests.

## Authors’ contributions

KB and MP participated in data collection. KB, EK, TP, MP and TV were involved in drafting the study. HW, KB and EK participated in data and statistical analysis and AT advised on the analytical approach. HW drafted the manuscript and all authors were involved in manuscript review and editing for intellectual content. All authors read and approved the final manuscript.

## Pre-publication history

The pre-publication history for this paper can be accessed here:

http://www.biomedcentral.com/1471-2458/14/82/prepub
